# Influence of moon and clouds on night illumination in two different spectral ranges

**DOI:** 10.1038/s41598-021-98060-2

**Published:** 2021-10-19

**Authors:** Jürgen Krieg

**Affiliations:** Fraunhofer Institute (IOSB) of Optronics, System Technologies and Image Exploitation, Gutleuthausstraße 1, 76275 Ettlingen, Germany

**Keywords:** Environmental sciences, Optics and photonics

## Abstract

The variable brightness of the night sky affects plants as well as animals and humans. However, knowledge about this variability is still insufficient. Outstanding questions regarding how significant the influence of the moon, clouds, and artificial lighting remain. To be able to make statements about these effects, measurements over a long period of time are necessary. Fraunhofer IOSB performs such measurements in the 380–780 nm photopic visual and 800–1700 nm shortwave infrared spectral range. As the latter is only marginally affected by artificial lighting, a comparison of the two bands deepens insight into the influence of artificial lighting. First analyses show that the moon is, as expected, the dominant light source in the night sky, especially during a full moon. Illuminance values up to 200 mlx and irradiance values up to 600 µW/m^2^ were measured in the visible and infrared respectively. The influence of clouds is more complicated. The measured intensities depend, among other things, on cloud cover and cloud altitude. When the night sky is overcast, the measured intensities can drop as low as 0.5 mlx and 0.5 µW/m^2^, respectively. These small values were measured during rainfall. The influence of artificial illumination is difficult to estimate, as intensities in the shortwave infrared decrease with increasing cloud cover, but increase in the visual.

## Introduction

How bright is the night sky? The answer to this question is important especially in times when artificial lighting at night (ALAN) is becoming more and more widespread. This has an ever-increasing impact on many areas of people’s live. The first thing in this context that comes to mind is astronomy. A century ago, observatories were located near cities, but today they are found far away on remote mountains. The illumination of the growing cities has made it impossible to make astronomical observations there. Astronomers have been carrying out measurements for many years in order to find out how bright the night sky is^1–3^.

However, the brightening of the night sky also has an ever-increasing impact on nature. Studies on animals have shown that ALAN not only affects behavior, but also acts at a molecular and physiological level^4–6^. In plants, changes were observed in phenology, growth and interaction with animals (herbivory, pollination)^7–9^. However, ALAN affects not only animals and plants but also humans. Inconsistencies in circadian rhythms, changes in melatonin secretion or an increase in breast cancer rates, for examples, are associated with it^10–12^.

Despite these factors, there is the insufficient knowledge about night illumination. How great is the variability over the night, over the lunar cycle, or over the year? How great is the influence of cloud cover or the artificial lighting to night illumination? Since, for example, the eyes of animals may exhibit maximum sensitivity in a different spectral range^13^ than human eyes, the investigations should be extended beyond the visual spectral range.

In order to obtain statistically relevant statements, long-term measurements are necessary. In the measurements performed so far, Sky Quality Meters (SQMs) and DSLR cameras were used in most cases^14–16^. In measurements performed at astronomical sites, astronomical equipment is used^17,18^. These measurements are usually limited to the zenith range and the visual spectral range. In addition, there is too little information on weather conditions and cloud cover during the measurements.

Fraunhofer Institute of Optronics, System Technologies and Image Exploitation (IOSB) measures and analyses night illumination levels in the visible (VIS) and shortwave infrared (SWIR) spectral range for night vision applications. In 2019, Fraunhofer IOSB began with long-term measurements near Storkow in Brandenburg, Germany^19^. An automated system simultaneously records illuminance in VIS, irradiance in SWIR, and cloud cover and environmental data each night from dawn to dusk. Breaks in the measurements occurred between mid-May and end of July, as the astronomical twilight lasted the entire night, and between the end of October 2019 until August 2020 due to technical problems and the SARS CoV 2 pandemic. Preliminary analysis of the data with respect to the influence of moonlight, clouds, and rain on night illumination will be presented in this paper.

## Results

### Influence of moonlight

**Figure 1:**
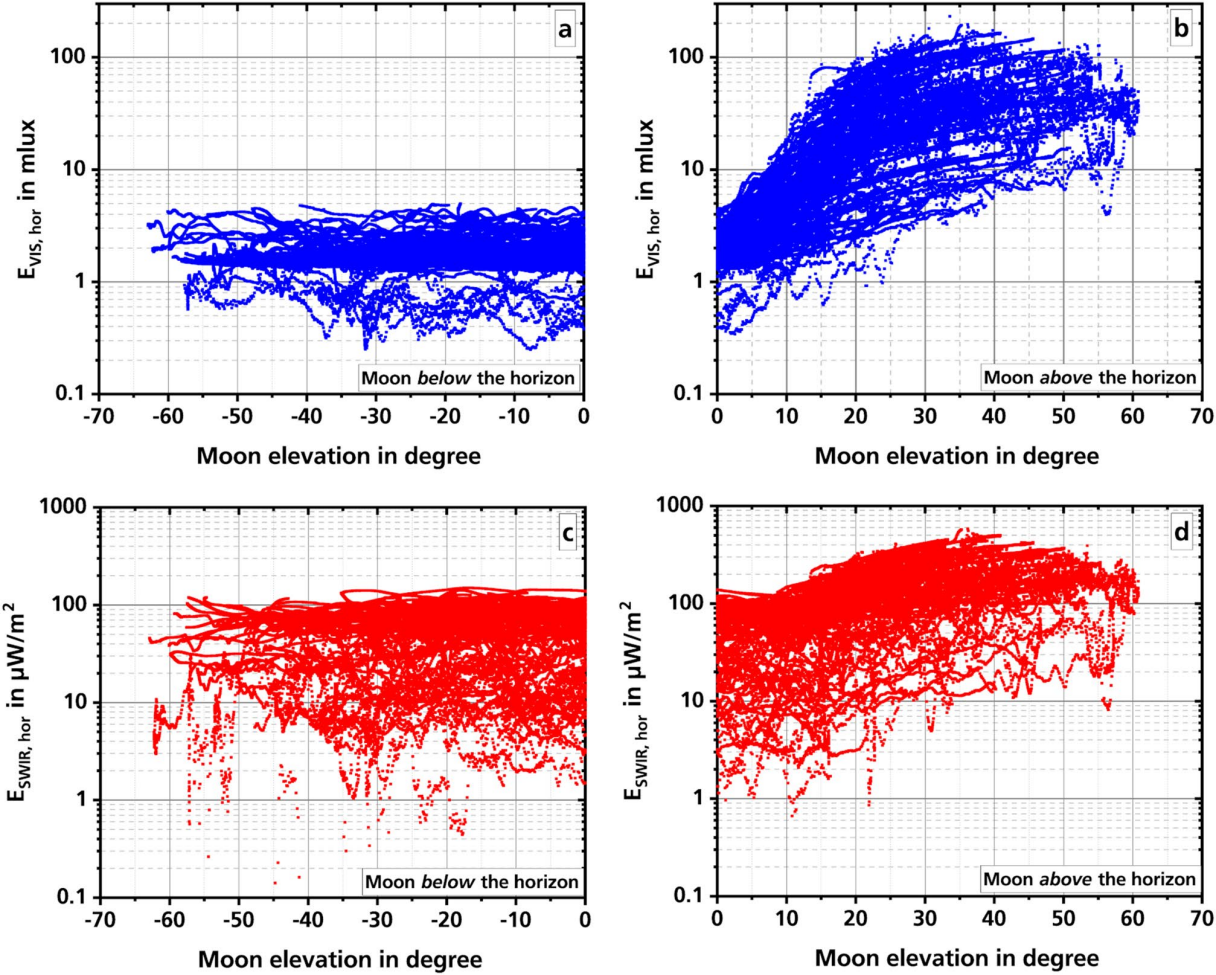
Dependence of night illumination on the lunar altitude in the VIS (**a**,**b**) and SWIR (**c**,**d**) spectral range. The graphs on the left side (**a**,**c**) show data when the moon was below the horizon, and on the right side (**b**,**d**) when the moon is above the horizon. The values are 1-min averages.

Aside from artificial lighting, the moon is the source of radiation that illuminates the night the most. Its irradiance is strongly dependent on the lunar phase and altitude above the horizon^18,20,21^. Figure 1 shows the large variability of night illumination as a function of the lunar altitude, measured with an illuminance (VIS) and an irradiance meter (SWIR). Details can be found in the “Methods” section.

Figure 1a shows the measurements in the VIS when the moon was below the horizon. The scatter of the measured values here is never greater than about a factor of 13, regardless of the altitude of the moon under the horizon. Most of the measured illuminance values are in the range between about 0.3 mlx and 4 mlx. Thus, much of the scatter in Fig. 1b can be attributed to the changing lunar phase, which is not shown here. The remaining factor of 13 could be due to variable illumination of clouds by artificial lighting. Also, the variable irradiation by nightglow^22^ as well as stars and planets will contribute as well.

Unsurprisingly, an increase in night illumination with increasing lunar altitude can be observed in the VIS (Fig. 1b). Of greater interest, however, is the wide dispersion of the values for each altitude. In some cases, the largest measured values are about a factor of 40 larger than the smallest measured values for the same lunar altitude.

**Figure 2:**
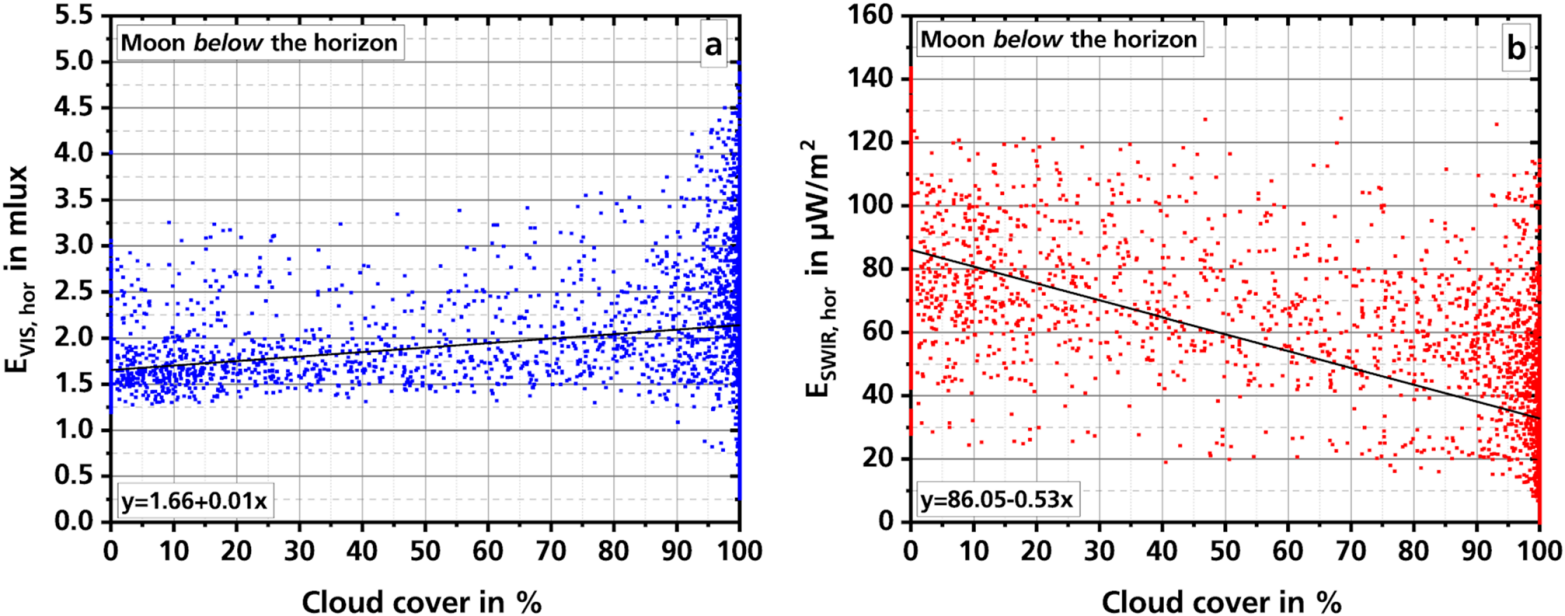
Dependence of night illumination in the two spectral ranges (**a**: VIS, **b**: SWIR) on cloud cover. The values are 7-min averages. The black lines in **a** and **b** show the result of a linear regression. The moon is below the horizon.

In the VIS, the measurements during the full moon phase gave the highest illuminance values at just below 200 mlx (Fig. 1b). They are thus about a factor of 400 above the minimum illuminance values of about 0.5 mlx, measured in a cloud covered night with no moon (Fig. 2a). The measured values are in good agreement with other published data^18,20^ from the center of Vienna, Austria and two locations in western Los Angeles, US. The reason that in both publications measured values are higher than those in this paper can be attributed to the fact that during our previous measurements the moon never passed its maximum attainable altitude above the horizon. This happens every year in December.

The two lower graphs in Fig. 1 shows the measurements in the SWIR spectral range with the moon below the horizon in graph c and above the horizon in graph d. Here too, the scatter of the measured values for an arbitrary lunar altitude is large, even with a factor of about 50 and more in graph d somewhat larger than in the VIS.

In the SWIR, the highest irradiance during the full moon phase were measured just below 600 µW/m^2^. In a cloud covered night with no moon only about 0.5 µW/m^2^ was measured (Fig. 2b). This is a much greater factor of 1200 compared to the factor measured in the VIS.

When comparing the brightness ranges for the two spectral ranges for the case that the moon is below the horizon (Fig. 1a,c), it should be noted that the illuminance in the VIS changes by about a factor of 13, but in the SWIR the irradiance changes by about a factor of 300. In the SWIR, only measured irradiance values between 0.5 and 150 µW/m^2^ are used, because the number of measured irradiance values smaller than 0.5 µW/m^2^ is still too low to make a significant statement. The reason for this great factor in the data of graph c is mainly the nightglow, which is much brighter in the SWIR (see Table 1 in^23^).

Another interesting point is when moonlight manifests in the illumination. For the SWIR the increase caused by the moon begins around 10 deg. moon elevation. This is distinctly higher than for the VIS where this increase already starts when the moon is slightly (app. − 2.5 deg.) below the horizon.

### Influence of clouds

Besides moonlight and ALAN, clouds have a major impact on the observation of night illumination. However, it is difficult to observe clouds during the night. Usually data from a nearby weather station^24,25^ or observations made with naked eye^26^ are used to determine the cloud cover and the altitude of the clouds. Rather rarely, ceilometer^27^ or radar^28^ are used. For the observations described here, measurements of cloud cover and cloud altitude were made using a Nubiscope (For details see “Methods” section). For the investigation of the influence of cloud cover and cloud altitude of the night illumination, the measured values of the illuminance and the irradiance meter are averaged over seven minutes. This corresponds to the measurement time of one scan of the Nubiscope. Figure 2 shows the dependence of night illumination in the two spectral ranges on cloud cover when the moon is below the horizon.

Graph a of Fig. 2 shows the measured values of the illuminance meter. It can be seen that with a cloud cover of less than 90% the measured illuminance values are never lower than about 1.25 mlx. Also, there are not very many data points above about 3.5 mlx. Only if the cloud cover rises to about 90% and more, the range in which measured illuminance values can be found increases to between 0.25 mlx and 5 mlx. A linear regression gives an intercept of 1.66 (0.02) and a slope of 0.01 (< 0.01), with standard errors in parentheses.

Graph b of Fig. 2 shows the data from the irradiance meter. The irradiance range from about 20 to about 120 µW/m^2^ with higher values between 30 and 140 µW/m^2^ for a clear sky and smaller values between close to zero and about 120 µW/m^2^ for nearly closed cloud cover. A decrease in irradiance with an increase in cloud cover can be seen. A linear regression yields a value for the intercept of 86.5 (0.59) and a slope of − 0.53 (0.01).

**Figure 3:**
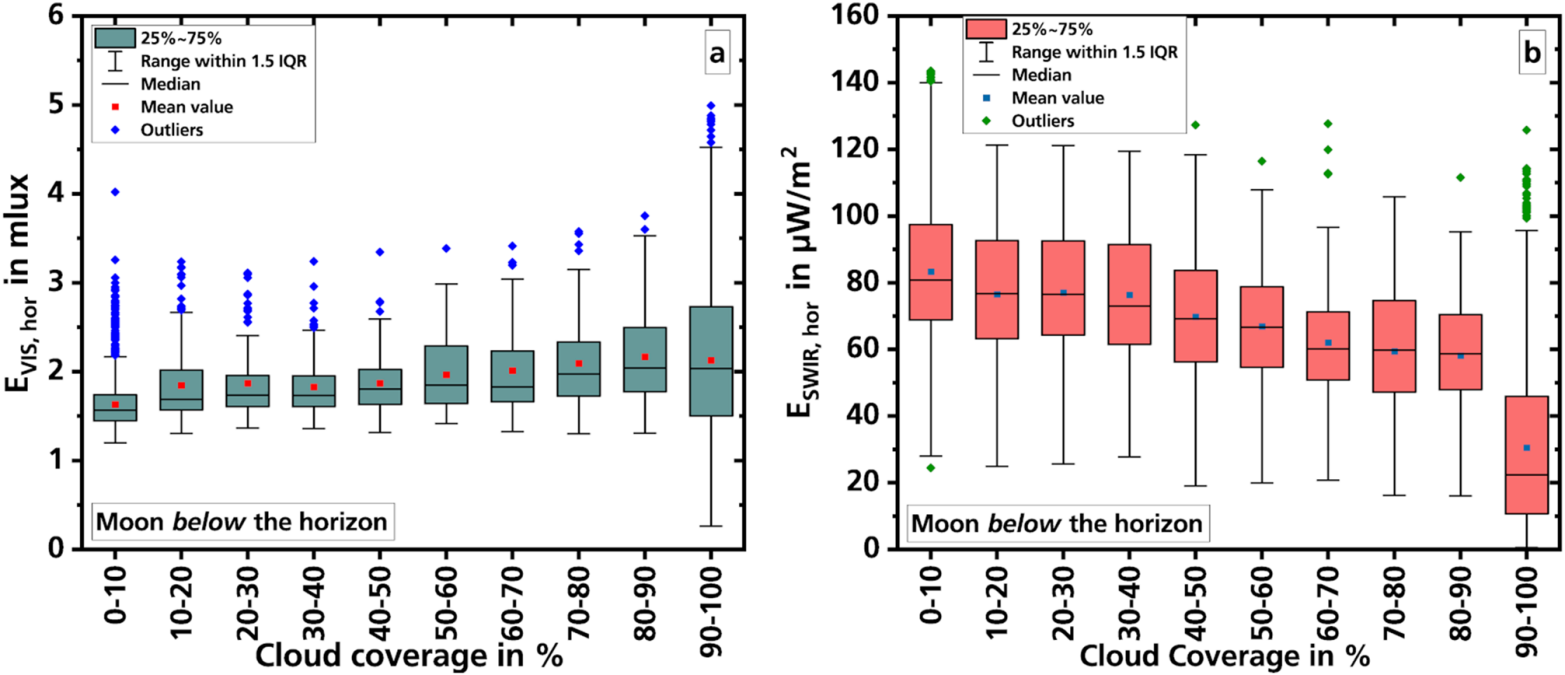
Dependence of night illumination in the two spectral ranges (**a**: VIS, **b**: SWIR) on cloud cover. The two graphs show boxplot diagrams where the measured values have each been averaged over 10 percentage points of cloud cover. The moon is below the horizon.

The large scatter of the measured values shown in Fig. 2 makes it difficult to identify a trend. In order to check the statements of the linear regressions performed, boxplot diagrams were calculated with the data (Fig. 3). For this purpose, the 7-min averages were distributed in such a way that they came to lie in the matching 10-percent interval of cloud cover according to the cloud cover degree measured at the same time.

Again, the VIS (Fig. 3a) shows a slight increase in illuminance with increasing cloud cover. In the SWIR (graph b), the downward trend can be confirmed. At the moment it is not known why in the SWIR the box for cloud cover between 90 and 100% is so significantly lower than the others.

Generally, a decrease in illuminance level with increasing cloud cover would be expected because clouds screen downwelling radiation. However, on the other hand, they reflect upwelling radiation. Thus, the difference between VIS and SWIR behavior may be explained by the different mixing of natural and artificial lighting. Although artificial light sources may also radiate in the SWIR by emission lines or by temperature radiation caused by heating during operation, this radiation is much smaller than the radiation created in the VIS. In contrast, without the moon, the night illumination in the VIS is known to be roughly one magnitude lower than in the SWIR (compare e.g.^29^). Thus, the high artificial radiation level easily increases the overall illuminance level when reflected by the clouds. For the SWIR the artificial illumination level is too low in contrast to the still transmitted nightglow and the expected decrease is observed.

**Figure 4:**
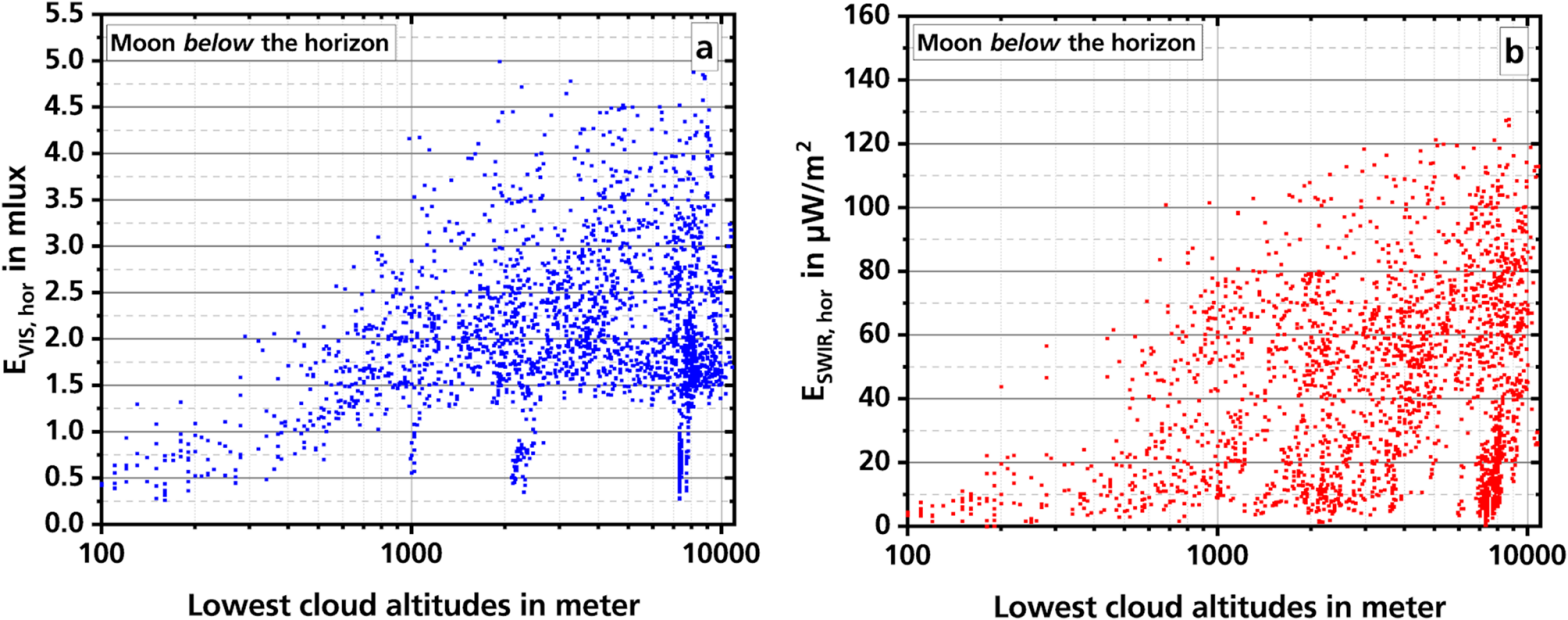
Dependence of night illumination in the two spectral ranges (**a**: VIS, **b**: SWIR) on the altitude of the lowest clouds. The moon is below the horizon. The values are 7-min averages.

The measured values are influenced by not only the cloud cover, but also the altitude of the clouds. The Nubiscope cannot measure the altitudes directly, but can calculate indirectly them from the measured values. As the two graphs in Fig. 4 show, in both spectral ranges an overall increase in the intensities can be observed with increasing cloud altitude.

In the VIS (Fig. 4a) the illuminance increases from an altitude of about 100 m up to about 1000 m relatively evenly. Above this, up to an altitude of about 11,000 m increasingly larger values are measured, but the smallest measured values remain almost on a plateau at about 1.5 mlx. The assumption for this behavior is that from a cloud altitude of about 500 m, more and more artificial lighting is reflected into the measuring range. The irradiation reaches its maximum at a cloud altitude of about 1000 m and then remains almost constant as the cloud altitude increases further. The reason for the lower illuminance values of less than 1.5 mlx for the data points at cloud altitudes of about 1000 m, 2200 m, and 7300 m is not known now. The data are from different measurement nights in April and May 2019 and October 2020. An assumption is that the glass dome protecting the detector was fogged with dew. The high values of relative humidity of 90% and more, which the weather station measured at the same time, agree with this. Another possibility is that wrong altitude values have been calculated by the Nubiscope at these times. But at the moment there is no concrete reason for this assumption.

Graph b of Fig. 4 shows the measurements in the SWIR. With increasing altitude of the lowest clouds, there are also larger irradiance values. However, there are very low intensities at any altitude. There is no plateau as seen in the VIS. This would fit to the already expressed assumption that clouds can strongly shield the radiation of the nightglow. Also, it appears that artificial lighting does not have a large effect on the measurements in the SWIR.

### Influence of rain

**Figure 5:**
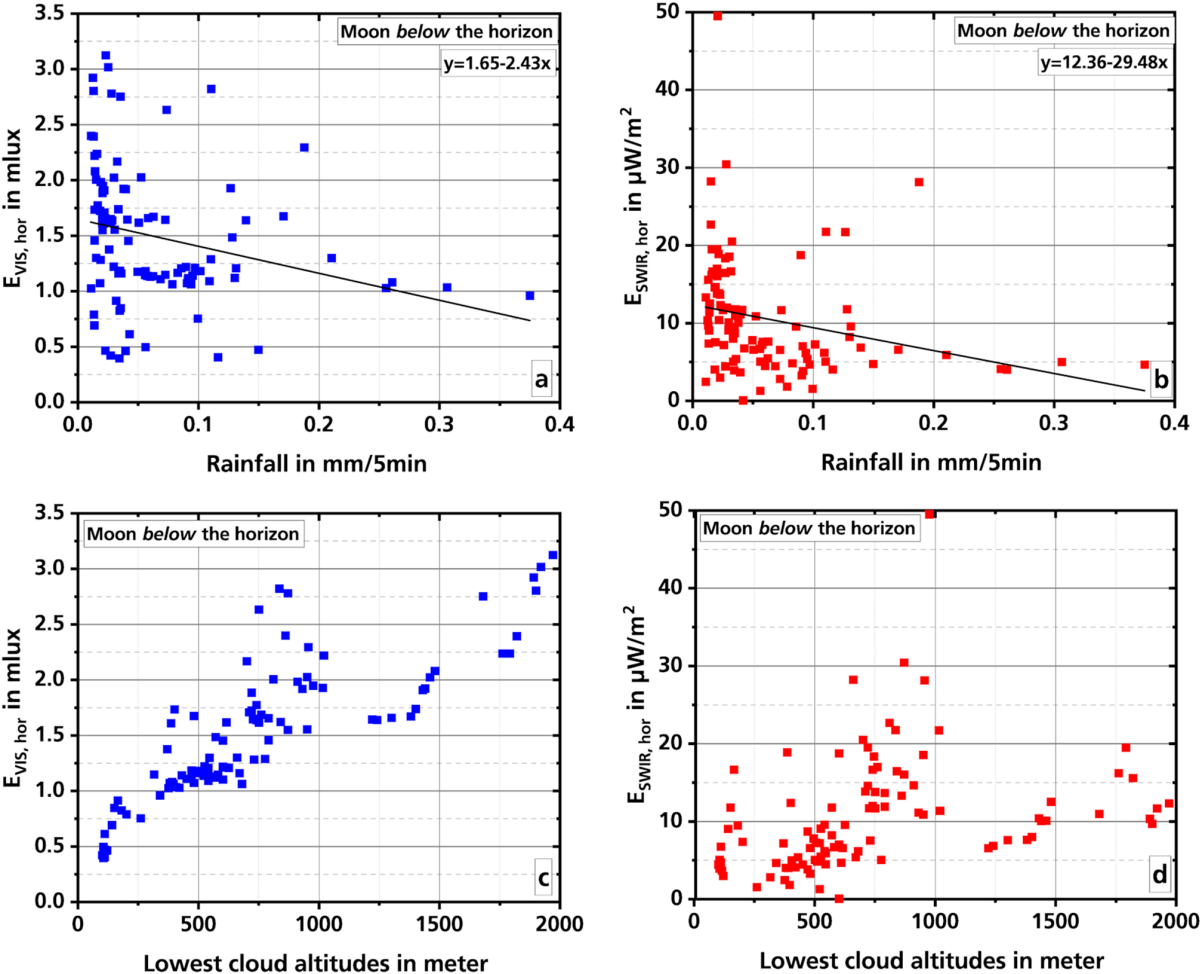
VIS and SWIR measured values during rainfall. The upper two graphs show the intensities in the VIS (**a**) and SWIR (**b**) as a function of rainfall. The lower two graphs show the altitude of the lowest clouds measured simultaneously for the VIS (**c**) and SWIR (**d**) data. The values are averages over five minutes. The black lines in **a** and **b** show the results of a linear regression. The moon is below the horizon.

The smallest values of the VIS and SWIR measurements were measured during rainfall. Figure 5 shows the result of these measurements (a, c: VIS, b, d: SWIR). During all rainfall cloud cover was greater than 99%. Since the weather station’s rain gauge only provides a value every five minutes, the data from the illuminance and the irradiance meter were also averaged over this time interval. In the graphs a and b of Fig. 5 a decrease in the measured values with increasing rainfall can be observed. A linear regression shows an intercept of 1.65 (0.08) and a slope of − 2.43(0.93) for the VIS measurements and values of 12.36 (0.99) and − 29.48 (10.96) for the SWIR measurements (standard errors in parentheses). However, the large standard errors for the slopes confirm the impression that this statement should be viewed with caution. The small number of only 101 measured values (August, September, and October 2019. No rain gauge measurements took place in 2020.) allows only an initial overview of the data at the moment.

While in the SWIR the high absorption of the incident radiation by the atmospheric water vapor can explain the decrease in irradiance, this is not the case in the VIS^30^. The data show here rather a correlation with the cloud altitude of the lowest clouds and less with the amount of rain. In graph c of Fig. 5, the altitude of the lowest clouds is plotted against the intensities in the VIS. The data reflect the correlation well. On the other hand, when comparing the intensities in the SWIR with the cloud altitude (graph d) there is no clear correlation.

## Discussion and summary

How bright is the night sky? This question from the introduction is not easy to answer, as the results presented here have shown. Many variables, some of them unknown in their magnitude, influence the night illumination. While the illumination by the moon, the stars and planets as well as the nightglow can be calculated or estimated, this is no longer possible for the contribution from artificial lighting. In addition, there is the influence of the earth’s atmosphere in which the incident radiation is scattered and absorbed. Also, clouds can reduce as well as increase the night illumination^31,32^.

Apart from artificial lighting, the moon is the brightest light source in the VIS with illuminance values up to almost 200 mlx during full moon phase. If the moon is below the horizon, minimum values of just 0.5 mlx were measured. In the SWIR, the measured irradiance values range from about 0.5 to 600 µW/m^2^. Our measurements also show that the influence of moon light is much greater in the VIS than in the SWIR. This is also shown by spectra recorded by Vatsia^29^ in the spectral range from 0.45 to 1.95 µm in clear nights at different lunar phases. The measured radiances between no moon and full moon differ in VIS by a factor of about 100. In the SWIR, on the other hand, the measured values differ by only about a factor of 2.

A much bigger problem is to determine how significant the influence of clouds on night illumination is. In addition to the cloud cover, the altitude of the clouds and their internal structure must also be considered. This requires not only measurements, but also modeling the latter not being part of this publication. To exclude the influence of moonlight, only measurements where the moon was below the horizon are considered in the following.

Our measurements show that the illuminance does not drop below about 1.25 mlx as long as the cloud cover is less than about 90%. In the SWIR, the irradiance does not fall below about 20 µW/m^2^. With increasing cloud cover in the VIS, a slight increase of the measured values can be observed, while the values in the SWIR decrease. Only at a cloud cover of more than 90%, the measured values decrease significantly, down to about 0.5 mlx in the VIS and to about 0.5 µW/m^2^ in the SWIR. The illuminance values are approximately in agreement with the values found by Jechow et al.^31^ during measurements in a rural area in Germany; over two nights only, one clear (1.10 mlx) and one overcast (0.68 mlx). Other measurements by the same author^33^, taken in Latvia, show values of only 0.22 mlx in overcast conditions, which is much less than our measurements. One reason for this could be the lower influence of artificial lighting on the measurements.

However, the measurements by Jechow et al.^31^ and those described in other publications can only be compared to a very limited extent with the measurements presented here, as measurement area, encountered weather conditions, and measurement equipment are different. Often a Sky Quality Meter or a DSLR camera is used. In contrast to the hemispherical coverage of the devices used in our measurements, the SQM is mostly used in the version with a lens (SQM-L). Then, according to the manufacturer, it has a field-of-view of only 20° (FWHM), without lens 84°, and no cosine correction^34^. In addition, it has a different spectral response than the human eye adapted illuminance meter used here. During the measurements, the SQM was generally aligned to the zenith^32,35,36^. Also, the measurement quantities of the instruments (SQM: mag/arcsec^2^, illuminance meter: lux) cannot be easily converted for comparison in a common set of units, thus complicating the problem. In order to be able to compare measurement results from the SQM and the illuminance meter, joint measurements with both types of instruments are planned for the future. A DSLR camera with fisheye can do both—luminance and illuminance (scalar and horizontal) measurements simultaneously and the green channel is at least close to the photopic band (closer than the SQM band). However, this method is not yet used for long term studies.

Independent of the measuring device, the data of Kyba et al.^35^ also show a slight increase of night illumination with increasing degree of coverage and a partly large scattering of the measured values. This increase is much stronger in the city (Berlin) than at the measurement site in the countryside (Fig. 4A in Kyba et al.). The increase in night illumination at the latter measurement site is in better agreement with the increase determined from our data. Natural the distance between a measurement site and a site of artificial lighting may influence the actual difference between clear and cloudy sky conditions as indicated by first measurements by Jechow et al.^37^.

In addition to cloud cover, the altitude of clouds above ground also has an effect on night illumination. It has been shown that with increasing cloud altitude night illumination also increases. At cloud altitudes below about 1000 m, illuminance is usually below 1.5 mlx. At higher cloud altitudes, illuminance can increase up to about 5 mlx. Above this cloud altitude, almost no measured values smaller than 1.5 mlx are found in our data. A similar behavior is also found in Ribas et al.^32^. There are no more measured values larger than about 22 mag/arcsec^2^ above a certain cloud altitude (larger values stand for a darker sky). However, this occurs at about 3000 m and thus clearly above the cloud altitude measured by us.

In the SWIR, the measured irradiance also increases with increasing cloud altitude, from near zero to about 140 µW/m^2^. However, very small measured values are found here for all cloud altitudes. A possible reason for the difference between VIS and SWIR could be the artificial illumination, which is less noticeable in the SWIR.

The smallest values in VIS as well as in SWIR were measured during rain. The data indicate that the measured intensity values decrease with increasing rainfall. However, with only 101 measured values this observation must be viewed with caution.

The measurement data as a whole exhibit large variability in night illumination for a measurement station in a rural area. In the VIS, the light of the moon is the most important light source in the night sky. It significantly influences the rhythm of life of flora and fauna—and thus also humans—in equal measure. This is quite different in cities, where artificial lighting is becoming more and more widespread. There, many nights are now as bright as full moon nights away from urban areas^38^. As a result, changes can be observed in animal and plant behavior, and growth, among other things^39,40^.

In contrast, radiation in the SWIR appears to have little or no effect on flora and fauna. Although there are fish, for example, whose eyes are sensitive to above 0.93 µm^41^, many living creatures, including humans, cannot register infrared radiation^42^. Studies of plants have shown that their growth can be enhanced by near-infrared radiation (up to one micrometer)^43^. However, both examples remain limited to the spectral range below about one micrometer. This means that they have very little impact on the SWIR spectral range, which only starts at about 0.9 µm. Despite this, the low artificial radiation in the SWIR is helpful in gaining insight into the influence of light pollution in the VIS by comparing VIS and SWIR behavior.

The results presented here can only be considered preliminary at this time. The measured values are too scattered to be able to make reliable statements. Initial results show the great influence of cloud cover on the measurements. Also, some results can be better explained if irradiation of artificial lighting into the measurement area is taken into account. However, a much larger amount of data is needed for a better estimation of the influence of the different environmental parameters. Therefore, it is planned to continue the measurements for at least one more year.

## Methods

### Measurement area

**Figure 6:**
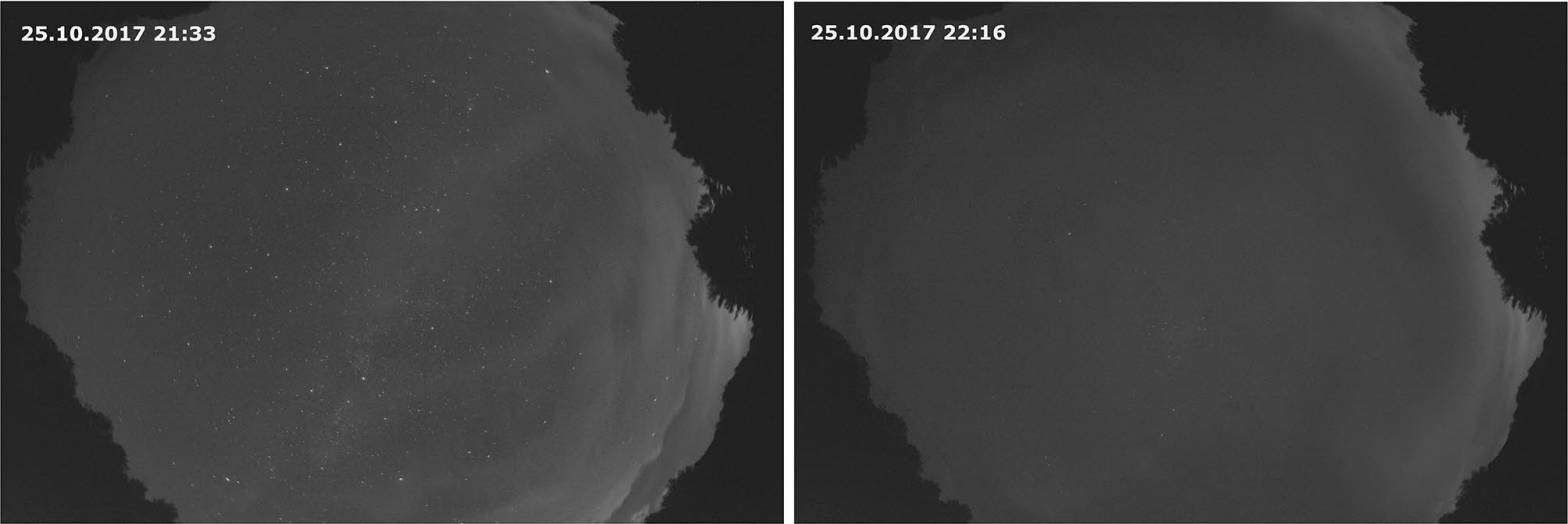
Effect of artificial stray light recorded with DSLR-Camera and fish eye lens during the Storkow test measurements 2017. Cirrus clouds enter the measurement area (left) and almost complete coverage (right). Times in CET. Reprinted with permission from^19^© The Optical Society).

Selecting a suitable measurement area was a difficult process as our application required remoteness from artificial lighting, ease of access, and security. These factors exclude each other to some extent: no artificial lighting typically means remote areas that can be difficult to reach and have poor security. Additionally, financial limitations had to be considered and our search was limited to areas in Germany or central Europe. Various sites were assessed^44,45^ and none was ideal from an artificial stray light point of view. The final compromise was a proving ground near Storkow, Brandenburg, Germany (52°11′ N, 13°56′ E, altitude 49 m). The small town is about 8 km away. Although located about 50 km southeast of Berlin, this area is in one of the darkest German regions. The light pollution map^46,47^ gives the following values for the zenith sky brightness: Proving ground: 21.6 mag/arcsec^2^ (0.247 mcd/m^2^), Storkow: 21.02 mag/arcsec^2^ (0.422 mcd/m^2^), and Berlin (Brandenburger Tor): 18.10 mag/arcsec^2^ (6.2 mcd/m^2^). These good conditions are confirmed by test measurements conducted there^45^. Figure 6 shows sky images from the test measurements with different cloud coverage. Stray light, especially at the horizon, is apparent. The effect of the stray light cannot be removed from the measured data and must be considered when analysing the data.

A forest clearing sufficiently large so as to avoid interference with the sensors from shadowing (such as that caused by trees in moonlight) was selected as the measuring area. Around the clearing, there is no artificial lighting at a radius of at least 1.5 km.

### Measurement equipment

**Figure 7:**
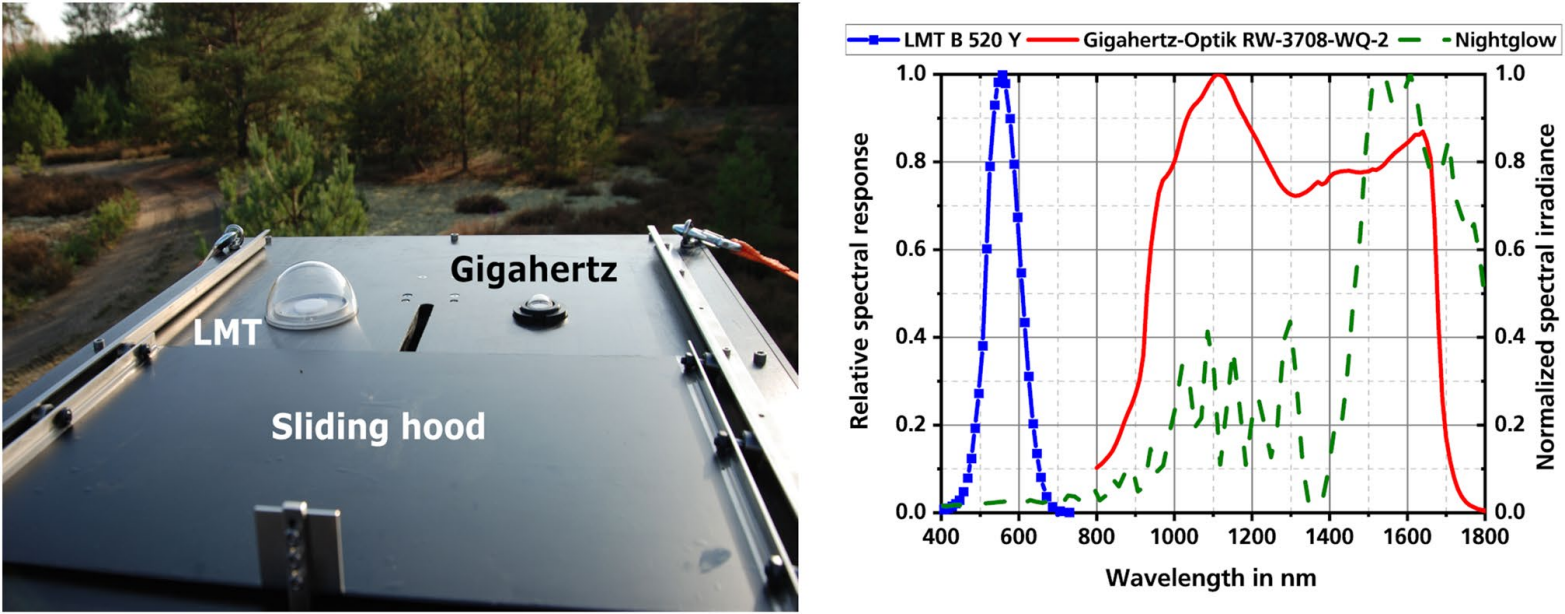
(**a**) The picture shows the housing with the two sensors under the glass domes. The sliding hood can be seen in the foreground. (**b**) Illuminance and irradiance meter relative spectral response in comparison to normalized starlight nightglow spectral irradiance^48^. ((**b**). Reprinted with permission from^19^ © The Optical Society).

The main measurement equipment used during the campaign is a LMT B520 illuminance and a Gigahertz-Optik RW-3708-WQ-2/P-9710-1 irradiance meter. The LMT B520 is a photopic response adapted, cosine corrected device with a minimum resolution of 10 µlx and 2.5 measurements per second with total error given as less than 7.5% by the manufacturer. The spectral response of the LMT B520 in comparison to the normalized starlight nightglow spectral irradiance is shown in Fig. 7b. The irradiance meter consists of the RW-3708-WQ-2 sensor head and the P-9710-1 display unit. It is also cosine corrected, with typical sensitivity of 40 nA/W/m^2^ and recording approximately two samples per second. The ± 7% device uncertainty is similar in magnitude to that of the illuminance meter. Spectral response is broadband, covering the typical SWIR-sensor spectral range from approximately 800–1700 nm (Fig. 7b). Both sensors are protected by a glass dome attached by the manufacturer. They are calibrated at least biyearly by the manufacturers with calibrations traceable to PTB, the national metrology institute of Germany.

The sensors are mounted in a common housing with the sensitive areas parallel to the ground (Fig. 7a). As a result of the measurements, a horizontal illuminance or horizontal irradiance is obtained. An automated sliding hood protect the sensors during the day. This setup is located approximately two meters above ground level to avoid interference from grass and bushes as well as provide protection from animals.

Standard metrological measurements are made to accompany the illumination characterization for the analysis (temperature, relative humidity, air pressure, global and diffuse radiation, visibility, etc.). Additionally, an IMK Nubiscope^49,50^ is used to measure cloud coverage for different altitude regions (low, middle, and high) with a scanning pyrometer to calculate the altitudes of clouds for these regions. The pyrometer detector can operate both day and night, and scans the whole sky automatically in 10 min intervals with each scan taking about seven minutes. Default settings are used for this measurement set.

All instruments are connected to a common PC for control and data storage with a remote data download option. The whole system is automated and autonomous. The meteorological station collects data both day and night, but the illumination sensors and Nubiscope operate only during nighttime (a few hours before astronomic dawn to a few hours after astronomic dusk). Solar panels are used to recharge the batteries to provide power, which provides flexibility in choice of location.

However, measurements will not be made from mid-May to the end of July since at this latitude astronomical twilight (Sun 18 deg. below the horizon) lasts the entire night with sunlight still illuminating upper parts of the atmosphere^51^. These breaks will are used for cleaning, recalibration, etc. For the analysis, data from the following months are used: 2019: March (21.–31.), April, May (1.–19.), August, September, October (1.–20.); 2020: August (4.–31.), September, October (1.–23.).

### Data processing

The acquired illumination data is preprocessed by averaging 1-min intervals. The resulting illumination plots for each night were visually examined for problems in data acquisition, strange illumination effects, etc. If the data from the Nubiscope is to be included, the illumination data are averaged over seven minute intervals, the approximate time required for one scan of the instrument. In both cases, the data is limited to times between the end (of the previous day) and before the beginning (of the current day) of astronomical twilight.

To examine the measurement values under different cloud cover levels, the data were distributed over 11 different areas of cloud cover, each of them 10 percentage points in size. Since the Nubiscope cannot determine the cloud cover of all cirrus clouds, this data is excluded here (moon above the horizon: 439 values, moon below the horizon: 434 values). They were also distributed according to whether the moon was above or below the horizon. Table 1 shows the distribution.

**Table 1:** Number of 7-min averages for the different cloud cover ranges, separated by lunar altitude (moon above or below the horizon).

Cloud cover (%)	0–9	10–19	20–29	30–39	40–49	50–59	60–69	70–79	80–89	90–100
Moon above horizon	1258	176	139	104	94	92	113	108	152	1611
Moon below horizon	1288	171	110	97	92	77	92	112	159	1546

For the preprocessing the software IDL (Interactive Data Language) was used. Statistical analyses were performed using ORIGIN software.
